# Efficient Genetic Safety Switches for Future Application of iPSC-Derived Cell Transplants

**DOI:** 10.3390/jpm11060565

**Published:** 2021-06-17

**Authors:** Julia Dahlke, Juliane W. Schott, Philippe Vollmer Barbosa, Denise Klatt, Anton Selich, Nico Lachmann, Michael Morgan, Thomas Moritz, Axel Schambach

**Affiliations:** 1 Institute of Experimental Hematology, Hannover Medical School, 30625 Hannover, Germany; dahlke.julia@mh-hannover.de (J.D.); schott.juliane@mh-hannover.de (J.W.S.); VollmerBarbosa.Philippe@mh-hannover.de (P.V.B.); Denise_Klatt@DFCI.HARVARD.EDU (D.K.); selich.anton@mh-hannover.de (A.S.); lachmann.nico@mh-hannover.de (N.L.); morgan.michael@mh-hannover.de (M.M.); moritz.thomas@mh-hannover.de (T.M.); 2 REBIRTH Research Center for Translational Regenerative Medicine, Hannover Medical School, 30625 Hannover, Germany; 3 Fraunhofer Institute for Toxicology and Experimental Medicine, 30625 Hannover, Germany; 4 Division of Hematology/Oncology, Boston Children’s Hospital, Harvard Medical School, Boston, MA 02115, USA

**Keywords:** iPSC, gene editing, safe harbor, lentiviral vector, safety switch, iC9, TK.007

## Abstract

Induced pluripotent stem cell (iPSC)-derived cell products hold great promise as a potential cell source in personalized medicine. As concerns about the potential risk of graft-related severe adverse events, such as tumor formation from residual pluripotent cells, currently restrict their applicability, we established an optimized tool for therapeutic intervention that allows drug-controlled, specific and selective ablation of either iPSCs or the whole graft through genetic safety switches. To identify the best working system, different tools for genetic iPSC modification, promoters to express safety switches and different safety switches were combined. Suicide effects were slightly stronger when the suicide gene was delivered through lentiviral (LV) vectors compared to integration into the AAVS1 locus through TALEN technology. An optimized HSV-thymidine kinase and the inducible Caspase 9 both mediated drug-induced, efficient in vitro elimination of transgene-positive iPSCs. Choice of promoter allowed selective elimination of distinct populations within the graft: the hOct4 short response element restricted transgene expression to iPSCs, while the CAGs promoter ubiquitously drove expression in iPSCs and their progeny. Remarkably, both safety switches were able to prevent in vivo teratoma development and even effectively eliminated established teratomas formed by LV CAGs-transgenic iPSCs. These optimized tools to increase safety provide an important step towards clinical application of iPSC-derived transplants.

## 1. Introduction

Induced pluripotent stem cells (iPSCs) harbor great potential as a cell source in cell and gene therapy to replace lost, damaged, non-functional or degenerated tissue. With the possibility to be generated from patient-specific adult cells and their capability to self-renew, iPSCs constitute a potentially unlimited source of autologous cells, which have fewer ethical restrictions for use than embryonic stem cells (ESs) [1]. Within the past decade, protocols to derive specifically differentiated, transplantable cell types from iPSCs have drastically increased in number and been improved in terms of the quality of generated cells. On this basis, clinical trials using iPSC-derived cells have already been launched. The very first of these was conducted in Japan in 2014 and treated macular degeneration with autologous iPSC-derived retinal pigment epithelium sheets [2]. Other attempts have focused on the establishment and transplantation of cardiomyocyte patches (NCT04396899), iPSC-derived mesenchymal stromal cells to combat graft versus host disease [3] or dopaminergic progenitors for transplantation into the corpus striatum of a Parkinson’s patient [4]. So far, none of the latter studies presented any unwanted side effects related to the transplantation. However, iPSCs are characterized by their capacity to form teratomas as an indicator of pluripotency, so that residual iPSCs or progenitors that remain in the differentiated culture imply a safety risk when transplanted into patients. Additionally, the respective differentiated cells might cause unforeseen toxicity upon transplantation. Therefore, the risk of potential tumorigenicity or other severe adverse events (SAE) currently limits the clinical applicability of iPSC-derived cells [5]. To address this obstacle and thus pave the way for broad clinical application of iPSC-derived transplants, there is a strong need to develop adequate and efficient purification methods and/or strategies to remove the graft, or certain cell types derived from the graft, in the case of any SAE.

Clinically applicable safety strategies have already been developed and demonstrated to be effective in the context of cell transplantation, most prominently in the field of chimeric antigen receptor (CAR) T cell therapy [6,7]. While some of these strategies are specific to T cells, the concept of so-called safety switches is ubiquitously applicable and involves a genetic element (the suicide gene) expressed within the cells, which causes cell death only in combination with a drug/inducer. The best-characterized and most widely used suicide gene is the *Herpes simplex virus thymidine kinase* (HSV-TK), which is able to phosphorylate nucleoside analogs, most commonly ganciclovir (GCV), that serve as prodrugs. Once triphosphorylated, these analogs cause cytotoxicity via inhibition of DNA synthesis upon their incorporation and consequently lead to cell death. Various generations of HSV-TK have been established [8,9] but the fully codon-optimized A168H-mutated version (TK.007) was demonstrated to be one of the most efficient variants due to a preserved catalytic activity for GCV and a decreased affinity for endogenous thymidine [10,11]. Another safety system is based on the inducible caspase 9 (iC9) suicide gene, which encodes for a fusion protein that consists of a modified human caspase 9 and the human FK506 binding protein (FKBP). In this system, apoptosis is induced upon conditional dimerization of iC9 that is facilitated through a chemical inducer of dimerization (AP1903 or AP20187) and activation of downstream pro-apoptotic effector molecules [7,12].

Since iPSCs can be efficiently modified via current gene transfer methods and cultivated as single-cell clones, it is possible to equip iPSCs with suicide switches. Afterwards, correctly modified clones can be characterized, expanded and used as an off-the-shelf cell source with improved safety. Different options are available for suicide gene delivery into iPSCs. As the suicide system needs to be active in the later graft, stable genetic introduction of an expression cassette encoding for the suicide gene into iPSCs is mandatory. Designer nucleases can serve to integrate the expression cassette, provided by a donor plasmid, into a safe harbor locus [13]. These loci are sites in the genome known to allow for predictable expression of newly inserted genetic elements without any alterations to the host genome that may pose risks. The *AAVS1* site in the *PPP1R12C* gene on chromosome 19q [14] is referred to as a safe harbor locus that allows stable transgene expression in iPSCs and their derivatives [15,16,17]. However, several studies provided evidence of a differential transgene silencing in the *AAVS1* locus [18,19]. Alternatively, transgene delivery to iPSCs can be accomplished through viral vector systems. With the hallmark of stable genomic integration, efficient iPSC transduction, and a large coding capacity, lentiviral (LV) vectors are very promising gene transfer tools. Early reports on the application of gammaretroviral vectors, which belong to the same vector family, in clinical trials to treat inherited disorders of the blood system, were overshadowed by the occurrence of vector-related SAE in the form of leukemia [20]. However, LV vectors were found to have a more favorable (semi)random integration pattern, and the vector safety was generally improved through introduction of the self-inactivating (SIN) design. Thus, LV vectors have emerged as one of the major technologies to introduce both marker and therapeutic genes into iPSCs [21].

For efficient expression of the suicide gene, a promoter that is active in the relevant cell type needs to be included in the expression cassette. Ubiquitously-expressing promoters provide high-level expression profiles in a large number of cell types, with the EF-1α [22], SFFV or EFS [23] promoters being frequently used in iPSCs. A short version of the CAG promoter (chicken beta actin, rabbit ß-globin promoter; CAGs) was shown to provide a robust and stable expression in pluripotent cells as well as in differentiated progeny [24,25]. Alternatively, for selective expression in iPSCs, promoters derived from pluripotency-specific genes can be used, such as the EOS (*Early Transposon promoter and Oct-4 (Pou5f1) and Sox2 enhancers*) promoter and the *Octamer-binding transcription factor 4* short response element (Oct4SRE). The EOS promoter was established in 2009 and characterized by higher transgene expression in pluripotent cells compared to other potential pluripotency-specific promoters, such as the Oct4 and Nanog promoters [26,27]. The Oct4SRE described by Vega et al. constitutes a shortened version of the Oct4 promoter, which, despite its small size of 967 bp, resulted in higher expression levels in iPSCs than the full-length version, which is approximately 4 kb [28].

Although previous studies have shown that suicide switch concepts confer a higher safety profile to iPSCs and their differentiated progeny in vitro as well as in vivo [6,29,30,31], no systematic comparison of different suicide genes, delivery methods and promoters has been performed in order to identify optimal combinations and by that provide the basis for an efficient and clinically applicable safety strategy for iPSC-derived cell products. Therefore, we tested, characterized and validated combinations for all three parameters and demonstrate efficacy and efficiency in in vitro assays as well as in an in vivo teratoma model, in which complete elimination of established iPSC-derived tumors was achieved upon activation of the safety switch.

## 2. Materials and Methods

### 2.1. Plasmids

#### 2.1.1. AAVS1 Donor Plasmids

The AAVS1 donor plasmid Donor_AAVS1.CAG.EGFP.RbGpa was kindly provided by Ulrich Martin (Hannover Medical School, Hannover, Germany) [32] and was modified through restriction digestion to replace the pre-installed CAG promoter and EGFP transgene with the CAGs promoter (also kindly provided by Ulrich Martin) and different transgene combinations used in this study. The transgene expression cassettes encoded either the far-red fluorescence protein Katushka2s (Kat2S) [33] only or Kat2S in combination with the fully codon-optimized and A168H-mutated TK.007 safety switch [10] and a truncated CD19 surface tag (∆CD19). For polycistronic expression of multiple transgenes, these were linked by 2A sites [34]. For termination of the transcript, a rabbit beta-globin poly A (RbGpA) sequence was installed, to generate AAVS1.CAGs.Kat2S.RbGpA (AAVS1 ctrl) and AAVS1.CAGs.TK.007.Kat2S.∆CD19.RbGpA (AAVS1 CAGs.TK). The expression cassettes of constructed donor plasmids were furthermore preceded by an element consisting of a splice acceptor (SA) and a puromycin resistance gene for selection of cells with correct integration into the AAVS1 locus. For homology-directed integration upon introduction of a double-strand break into the first intron of *PPP1R12C* (AAVS1) on chromosome 19, two 750 bp homology arms were included in the donor plasmid flanking the sequences to be integrated.

#### 2.1.2. Lentiviral Vectors

Safety switch and promoter combinations were cloned with enzymatic restriction digestion into a lentiviral vector backbone. The 3rd generation lentiviral SIN vector [35] contained the small chromatin opening CBX3 element [36] upstream of the respective promoter to prevent potential silencing of the inserted transgene(s). The following promoter/safety switch combinations were incorporated: CAGs.TK007.Kat2S.∆CD19, CAGs.iC9.Kat2S.∆CD19, Oct4SRE.iC9.Kat2S.∆CD19 and EOS.iC9.Kat2S.∆CD19. A vector carrying CAGs.Kat2S was used as a control. Downstream of the transgene cassette, a woodchuck hepatitis virus post-transcriptional regulatory element (wPRE) was included in the 3′ untranslated region to enhance vector titers and transgene expression [37]. All cloning details are available upon request.

### 2.2. Cell Cultivation

A previously generated human CD34^+^-derived iPSC line was used [38] and cultured in Dulbecco’s modified Eagle’s medium (DMEM)/F12 supplemented with 20% knock-out serum replacement (KSR), 1% non-essential amino acids (NEAA; all Gibco, Karlsruhe, Germany), 2 mM L-glutamine (Biochrom AG, Berlin, Germany), 100 U/mL penicillin, 100 µg/mL streptomycin (both from PAN Biotech, Aidenbach, Germany), 100 µM β-mercaptoethanol (Sigma Aldrich, Seelze, Germany) and 20 ng/mL human basic-fibroblast growth factor (bFGF; kindly provided by the Institute for Technical Chemistry, Leibniz University Hannover, Hannover, Germany) on γ-irradiated C3H murine embryonic feeder cells (MEFs; kindly provided by the MPI for Molecular Biomedicine, Muenster, Germany). For weekly accomplished passaging, MEFs were plated on 0.1% bovine gelatin (Sigma Aldrich)-coated dishes in DMEM low glucose, 15% heat-inactivated fetal bovine serum (FBS; both from PAN Biotech), 2 mM L-glutamine, 1% NEAA, 100 U/mL penicillin, 100 µg/mL streptomycin and 100 µM β-mercaptoethanol. To passage of iPSC cultures, the medium was supplemented with 10 µM Y-27632 (Rho-Associated Protein Kinase Inhibitor; Tocris, Bristol, UK) from 1 h before until the day after splitting.

Adherent human embryonic kidney (HEK) 293T cells were cultivated in DMEM (Biochrom AG) containing 10% heat-inactivated FBS, 100 U/mL penicillin, 100 µg/mL streptomycin, and 1 mM sodium pyruvate (PAN Biotech), and passaged every 2–3 days with trypsin (PAN Biotech).

### 2.3. TALEN induced AAVS1 Safe Harbor Targeting

iPSCs were transfected using Lipofectamine Stem Transfection Reagent or Lipofectamine 3000 (Thermo Fisher, Darmstadt, Germany) as per the manufacturer’s instructions. In brief, the day before lipofection, 0.5–1 ∗ 10^5^ iPSCs were seeded onto Geltrex precoated 24-well plates (Sarstedt, Nümbrecht, Germany) in feeder pre-conditioned medium supplemented with 10 µM Y-27632. For induction of a double-strand break followed by homology-directed integration into the AAVS1 locus within the *PPP1R12C* gene, plasmids encoding for transcription activation like effector nucleases (TALEN; kindly provided by Toni Cathomen, University Medical Center Freiburg, Freiburg, Germany) [39] and the AAVS1 donor plasmid were co-transfected. For this, the plasmids were combined with the recommended amount of lipofectamine and the lipofection mixture was added to the cells in the presence of 10 µM Y-27632. The cells were cultivated without antibiotics for two days. Then, puromycin selection (0.3 µg/mL; Invivogen, San Diego, CA, USA) was performed for 2 weeks to select for correctly targeted iPSC clones. Prior to use for further experiments, single colonies were picked three consecutive times.

### 2.4. Verification of Correctly Targeted iPSC

To identify correctly targeted iPSC clones and distinguish mono- vs. biallelic integration patterns, PCR amplification of the DNA integrated into the AAVS1 locus was performed from isolated genomic DNA with the Phire HS II polymerase (Thermo Fisher, Darmstadt, Germany) using the following primers: wildtype: AAVS1_wt_fw: GACAGCATGTTTGCTGCCTCC, AAVS1_w_rev: GGATCCTCTCTGGCTCCATCG; 5′Integration: AAVS1_5′Int_fw: CTGCTTTCTCTGACCTGCATTC, AAVS1_5′Int_rev: GGGCTTGTACTCGGTCATCTCG; 3′Integration: AAVS1_3′Int_fw: CCAAGTTCGGGTGAAGGCCC, AAVS1_3′Int_rev: AAGCCTGAGCGCCTCTCCTG. For detection of off-target integrations, Phusion HSII Green PCR mix (Thermo Fisher, Darmstadt, Germany) was used with the primer pairs specific for the 5′ end (AAVS1_5′off: GGGTGTCGGGGCTGGCTTAAC and AAVS1_5′Int_rev: GGGCTTGTACTCGGTCATCTCG) and for the 3′ end of the plasmid (AAVS1_3′off_fw: GTGAGAATGGTGCGTCCTAGG and AAVS1_3′off_rev: TATAGTCCTGTCGGGTTTCGCC).

### 2.5. Production of Lentiviral Particles

Lentiviral SIN vector particles were produced as per standard protocol, involving transient transfection of all required split-packaging system components using calcium phosphate transfection, as described elsewhere [40]. In brief, 1 * 10^7^ HEK 293T cells were treated with a transfection mix containing 10 μg of the lentiviral vector transfer plasmid [35], 5 µg of the plasmid pRSV-Rev (kindly provided by T. Hope, Northwestern University Chicago, IL, USA), 12 μg of the plasmid pcDNA3.GP.4xCTE, encoding for lentiviral Gag-Pol proteins, and 1.5 µg of the expression plasmid pMD.G, encoding for the vesicular stomatitis virus G envelope protein (VSVg) (Plasmid Factory, Bielefeld, Germany). The DNA was precipitated with 0.1 M CaCl_2_ (Merck, Darmstadt, Germany) in 2× HEPES-buffered saline. To enhance the transfection efficiency, 10 mM HEPES (PanBiotech) and 25 μM chloroquine (Sigma Aldrich) were added to the medium. The produced lentiviral particles were harvested 36 h and 48 h after transfection, filtered through a Millex-GP 0.22 µM filter (Millipore, Darmstadt, Germany), and concentrated 100× through ultracentrifugation for 2 h at 4 °C and 82,740× *g*.

### 2.6. Transduction of iPSCs with Lentiviral Vectors

Prior to transduction, iPSCs were incubated for 1–2 h with 10 µM Y-27632. To generate single cell suspensions, the cells were detached with StemPro^®^Accutase^®^ (Gibco) and 3 * 10^4^ iPSCs were transduced at a multiplicity of infection (MOI) of 3–10 in the presence of 4 µg/mL protamine sulfate (Sigma Aldrich). After 1 h of incubation under normal cell culture conditions, the suspension was plated onto feeder-coated cell culture plates to allow single-cell colonies to grow. Transduction efficiencies were determined by flow cytometric analysis with a CytoFLEX Flow Cytometer (Beckmann Coulter, Krefeld, Germany) and FlowJo software (LLC, Ashland, OR, USA). Prior to use in further experiments, the cells were sorted for Kat2S expression, seeded at a low density (1 × 10^4^) onto feeder-coated 10-cm dishes and single colonies were picked three consecutive times.

### 2.7. Embryoid Body-Based Differentiation into Macrophages

Embryoid body (EB)-based myeloid differentiation of iPSCs was performed as already described by our group [41]. In brief, iPSC cultures were allowed to grow densely, then treated with Dispase (Roche, Darmstadt, Germany) and detached colonies were suspended in Knock-out DMEM (Thermo Fisher Scientific, Darmstadt, Germany), 20% KSR, 2 mM L-glutamine, 2 mM NEAA, 100 U/mL penicillin, 100 µg/mL streptomycin and 100 µM β-mercaptoethanol, supplemented with 10 μM Y-27632. The cell-aggregates were transferred to suspension plates and placed on a Celltron orbital shaker (Infors HT, Einsbach, Germany) at 80 rpm under standard culture conditions (37 °C, 5% CO_2_) for about 5 days. Aggregated EBs were manually transferred to X-VIVO 10 medium (Lonza, Basel, Switzerland), supplemented with 100 U/mL penicillin, 100 µg/mL streptomycin, 50 µM β-mercaptoethanol, 25 ng/mL human interleukin-3 (IL-3) and 50 ng/mL human macrophage colony-stimulating factor (M-CSF) (both PeproTech, Hamburg, Germany) on adherent tissue culture plates (TPP, Trasadingen, Switzerland). After approximately one week, and, from then on, every 3–5 days, myeloid cells produced by myeloid cell-forming complexes (MCFC) were harvested from the supernatant and terminally differentiated into macrophages for about 5 days in Roswell Park Memorial Institute 1640 medium (RPMI 1640; PAN-Biotech), 10% FBS, 2 mM L-glutamine, 100 U/mL penicillin, 100 µg/mL streptomycin and 50 ng/mL M-CSF.

### 2.8. Cytospin Analysis

The morphology of differentiated macrophages was investigated by sedimentation of the cells on glass slides by cytocentrifugation (Cytospin 4 centrifuge, Thermo Fisher, Darmstadt, Germany) for 10 min at 400× *g*. Overnight air-dried glass slides were stained in May-Grünwald and Giemsa staining solutions (both Sigma Aldrich) according to the manufacturer’s instructions. Pictures were acquired with an Olympus BX51 microscope using Cell^F software (Olympus, Hamburg, Germany).

### 2.9. Flow Cytometry

Surface marker expression was determined by staining the cells against TRA-1-60, CD14, CD163, CD45 (all BioLegend, San Diego, CA, USA), CD11b (eBioscience, San Diego, CA, USA) and CD19 (Miltenyi Biotec, Bergisch Gladbach, Germany). Gating was performed according to isotype controls and non-expressing cells of the DAPI (Sigma Aldrich)-negative cell population. Analysis was done with the CytoFLEX Flow Cytometer (Beckman Coulter) and FlowJo software (LLC).

### 2.10. RT qPCR

Total RNA was purified from cell pellets with the QIAGEN RNeasy Mini Kit followed by cDNA production using the QuantiTect Reverse Transcription Kit according to the manufacturer’s instructions (both Qiagen, Hilden, Germany). Quantification of gene expression was assessed using the QuantiTect SYBR Green RT PCR Kit (Qiagen) and a StepOnePlus Real-Time PCR machine (Applied Biosystems, Darmstadt, Germany). Expression levels of *NANOG* and *OCT4* were determined with specific primers (NANOG 5′- TCACACGGAGACTGTCTCTC-3′ and 5′- GAACACAGTTCTGGTCTTCTG-3′ and OCT4 5′-CCTCACTTCACTGCACTGTA-3′ and 5′-CAGGTTTTCTTTCCCTAGCT-3′) [42] and calculated using the ΔΔCt method, related to the expression of the *β-ACTIN* housekeeping gene (Primer: 5′ CCTCCCTGGAGAAGAGCTA 3′ and 5′ TCCATGCCCAGGAAGGAAG 3′).

### 2.11. Vector Copy Number (VCN) Determination

To determine the number of vector copies integrated into the host cell genome, quantitative real time PCR was performed on genomic DNA (purified with Blood gDNA extraction kit, Qiagen) using ABI Taqman Fast Advanced Master Mix (Thermo Fisher) and a StepOnePlus Real-Time PCR machine. The vector copies per cell were determined by detecting the quantity of *wPRE* present in the integrated vector in relation to the abundance of the constitutively expressed host gene *PTBP2* (polypyrimidine tract-binding protein 2). Primers and probes used for this experiment: wPRE_fw: GAGGAGTTGTGGCCCGTTGT, wPRE_rev: TGACAGGTGGTGGCAATGCC; wPRE_probe: CTGTGTTTGCTGACGCAAC labeled with 5′-FAM, 3′-BHQ1. PTBP2_fw TCTCCATTCCCTATGTTCATGC, PTBP2_rev: GTTCCCGCAGAATGGTGAGGTG; PTBP2_probe: ATGTTCCTCGGACCAACTTG labelled with 5′-JOE, 3′-BHQ1.

### 2.12. In Vitro Ablation of Transgenic Cells

Viability assays were performed to assess the ablation of gene-modified cells in vitro. For that, 1 ∗ 10^4^ (iC9) or 5 ∗ 10^3^ (TK.007) transgenic cells were seeded per well onto white opaque 96 well plates coated with Geltrex (Gibco). The next day, the cultures were treated with different concentrations of GCV (InvivoGen, San Diego, CA, USA), AP20187 (Takara, Saint-Germain-en-Laye, France) or vehicle (0.9% NaCl or 0.4% ethanol). GCV-treated cells were incubated for 5 days prior to final readout, whereas AP20187 treated cells were analyzed 24 h post-drug-administration using the CellTiterGlo^®^ 2.0 cell viability assay (Promega, Mannheim, Germany) and the TriStar2 S LB 942 multimode microplate reader (Berthold, Bad Wildbad, Germany) or SpectraMax^®^ParadigmTM Multi-Mode Microplate detection platform (Beckmann Coulter).

### 2.13. Animals

NOD.Cg-*Prkdc*^scid^ Il2rg^tm1Wjl^/SzJZtm (NSG) mice were bred and maintained at the animal facility at Hannover Medical School in a pathogen-free environment. All animal experiments were performed according to institutional guidelines and federal law under the control of the Lower Saxony State Office for Consumer Protection and Food Safety (LAVES).

### 2.14. In Vivo Ablation of Transgenic Cells

For teratoma-based in vivo ablation assays, the development of teratomas was induced in 7–10-week-old NSG mice using an established protocol [43]. Briefly, 3 * 10^6^ transgenic iPSC, pretreated with 10 µM Y-27632 for 1–2 h, were suspended in cultivation medium supplemented with 20 µM Y-27632. The suspension was mixed with an equal volume of Corning^®^ Matrigel^®^ Basement Membrane Matrix (Corning, Corning, NY, USA) and subcutaneously injected into both flanks of the mice. The safety switches were induced at different time points, either immediately after iPSC inoculation or after 2 (iC9) or 3 (TK.007) weeks, when palpable teratomas had developed. For that, mice were intraperitoneally injected daily with either GCV (TK.007-transgenic cells), AP20187 (iC9-transgenic cells) or vehicle (0.9% NaCl or AP20187 buffer (4% ethanol, 10% PEG-400, 1.7% Tween)) for three days. Three to six weeks after inoculation, or when the tumors had reached a diameter of 15 mm, the mice were sacrificed and teratomas dissected. The tumors were subsequently fixed in 4% paraformaldehyde (Electron Microscopy Sciences, Hatfield, PA, USA), embedded into paraffin, cut into 3 µM slices and analyzed after HE staining with an Olympus BX51 microscope and Cell^F software. During the experiment, the tumor diameters were assessed with a caliper once a week; cystic fluid-filled teratomas were excluded.

### 2.15. Statistical Analyses

Statistical analyses were accomplished using GraphPad Prism software (GSL Biotech, Chicago, IL, USA). All graphs represent the mean ± standard deviation (SD). For statistical comparison, two-way ANOVA was used with Bonferroni’s multiple comparisons test and significances were determined as ns = not significant, * *p* ≤ 0.05, ** *p* ≤ 0.01, *** *p* ≤ 0.001, **** *p* ≤ 0.0001.

## 3. Results

### 3.1. Establishment of iPSC Lines Stably Expressig Safety Switches

**Figure 1:**
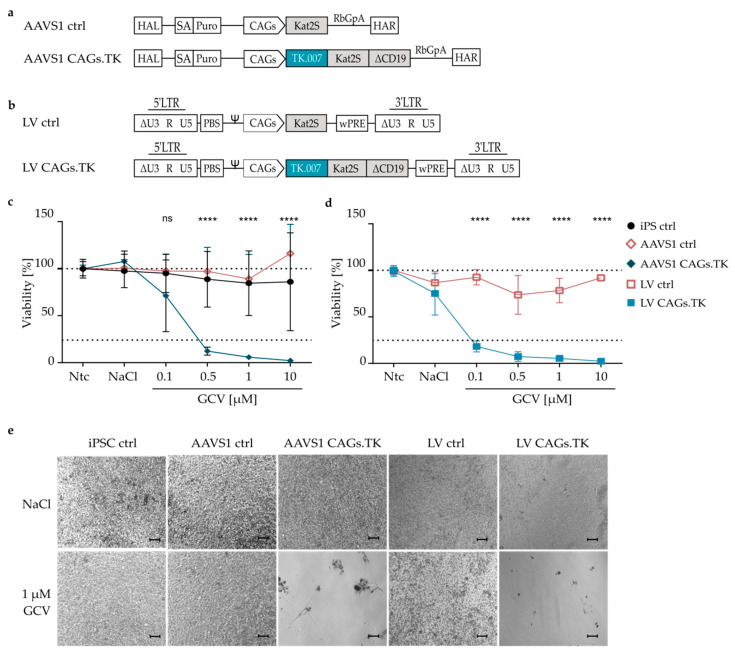
Efficient Ganciclovir-dependent in vitro ablation of TK.007 transgenic iPSC. (**a**) Plasmid donors for TALEN-mediated integration of the indicated cassette flanked by homology arms into the AAVS1 safe harbor locus on chromosome 19. The control (AAVS1 ctrl) expresses the far-red fluorescence protein Kat2S from the CAGs promoter, whereas the AAVS1 CAGs.TK additionally encodes for the TK.007 safety switch and the ΔCD19 surface tag. HAL = left homology arm; SA = splice acceptor; Puro = puromycin resistance gene; RbGpA = rabbit beta-globin poly-A termination signal; HAR = right homology arm. (**b**) Architecture of the SIN lentiviral control vector (LV ctrl) encoding for Kat2S and the vector encoding for the TK.007 safety switch and the Kat2S and ΔCD19 markers (LV CAGs.TK). LTR = long terminal repeat; ∆U3 = deleted enhancer/promoter region; R = repeat region within the LTR; Ψ = PSI, packaging signal; PBS = primer binding site; wPRE = woodchuck post-transcriptional regulatory element. (**c**,**d**) Percentage of viability relative to non-treated control (Ntc) as determined by CellTiterGlo^®^ 2.0 cell viability assay. Unmodified iPSC (iPSC ctrl) and transgenic iPSC (AAVS1 ctrl, AAVS1 CAGs.TK, LV ctrl and LV CAGs.TK) were treated for 120 h with GCV at the indicated concentrations, treated with the vehicle (NaCl) only or left untreated. iPSC ctrl N = 5–7, AAVS1 ctrl N = 3, AAVS1 TK N = 3, LV ctrl: N = 2–3, LV CAGs.TK N = 2–3. Dotted lines indicate 100% or 25%. (**e**) Microscopy images of the indicated iPSC cultures treated with vehicle (NaCl) or with 1 µM GCV upon 120 h of treatment. Scale bar 100 µm. **** *p* ≤ 0.0001.

Future clinical translation of iPSC-derived cell products will largely profit from the establishment of mechanisms to remove the graft or certain cell populations derived thereof in the case of SAE. Modification of the cells with genetically anchored safety switches appears to be a promising strategy in this context. In order to establish an optimal platform, we systematically compared and validated different options with respect to the main parameters critical for the success of a genetic safety switch system defining the best working combination: (i) the method used for stable introduction of the genetic cassette to express the safety switch; (ii) the nature of the safety switch; and (iii) the promoter that drives expression of the safety switch. In the first set of experiments, we compared two different methods that allow stable integration of an expression cassette into iPSCs, namely, targeted integration into a safe harbor locus, the *AAVS1* locus, versus introduction through LV vector technology. As a safety switch, the fully codon-optimized A168H mutant of the TK.007 transgene was chosen as its functionality in other contexts was previously demonstrated and it is approved for clinical application [44,45]. The expression cassettes used in both settings were identical, and employed the reportedly strong and ubiquitously-expressing CAGs promoter to drive expression of TK.007 along with the two selection markers: the far-red fluorescence protein Kat2S and a truncated version of the cluster of differentiation surface protein 19 (ΔCD19). Constructs only expressing Kat2S from the CAGs promoter served as control (ctrl) (Figure 1a,b). For safe harbor integration, we used the well-characterized TALEN-based gene editing system and components targeting the *AAVS1* locus [39]. The donor plasmid transferring the expression cassette to be integrated into this locus also contained a 5′ splice acceptor (SA) followed by a puromycin resistance cassette to allow for the selection of correctly targeted cells. Puromycin-selected cell clones were analyzed for mono- or biallelic, as well as for off-target integrations of the donors through a PCR-based approach (Figure S1a), which identified a clone with biallelic integrations without any off-target integration that was used for all further experiments. LV vector-transduced iPSCs were characterized for their mean VCN, and cells with a VCN of 1.7 were chosen for the control vector (LV ctrl) and those with a VCN of 0.7 for the safety switch vector (LV CAGs.TK, Figure S1b), to remain in a range of integrated copies of the expression cassette comparable to the AAVS1 setting. Importantly, genetic iPSC modification through the selected approaches did not affect pluripotency, as the cells had mRNA levels of pluripotency markers *OCT4* and *NANOG* comparable to untreated iPSC controls and embryonic stem cells, and expressed the pluripotency marker TRA-1-60 in the entire population as confirmed using flow cytometry (Figure S1c,d). Flow cytometric analyses further revealed the installed expression cassettes to be active in all genetically modified cells, with stable expression of Kat2S (all constructs) and ΔCD19 (encoded by suicide switch constructs only). Of note, the LV vector-transduced cells demonstrated higher expression levels of these markers as compared to AAVS1-modified cells (Figure S1e).

### 3.2. Efficient Ablation of TK.007-Transgenic iPSC upon GCV Treatment

After the characterization of genetically modified iPSCs, we directly compared the efficiency of GCV-induced and TK.007-mediated ablation of the cells modified through the two different transfer systems. Unmodified iPSC (iPSC ctrl) and control cells expressing Kat2S but not TK.007 (AAVS1 ctrl, LV ctrl), survived GCV treatment with the percentage of live cells remaining above 70%, although the variability among replicates was high and some minor toxic effects were observed at high GCV doses (Figure 1c). However, variability among replicates was also observed in vehicle-treated cultures that were not treated with GCV. Importantly, upon 120 h of treatment with different amounts of GCV, TK.007-expressing iPSCs (AAVS1 CAGs.TK, LV CAGs.TK) were efficiently ablated, achieving near complete elimination of all cells in the culture with 10 µM GCV. Interestingly, at a low dose of 0.1 µM GCV, LV vector-transduced cells were more efficiently eliminated than AAVS1-transgenic counterparts, with 80% (LV) and 30% (AAVS1) of induced cell death (Figure 1c,d). In contrast, at higher GCV doses of 1 µM, differences were less pronounced as an almost complete elimination of the treated cells was achieved in both settings (Figure 1e). This demonstrates functionality and a high efficiency of the TK.007/GCV-based safety switch concept in vitro and the suitability of both AAVS1-targeting approaches and LV transfer for stable introduction of the genetic safety switch.

**Figure 2:**
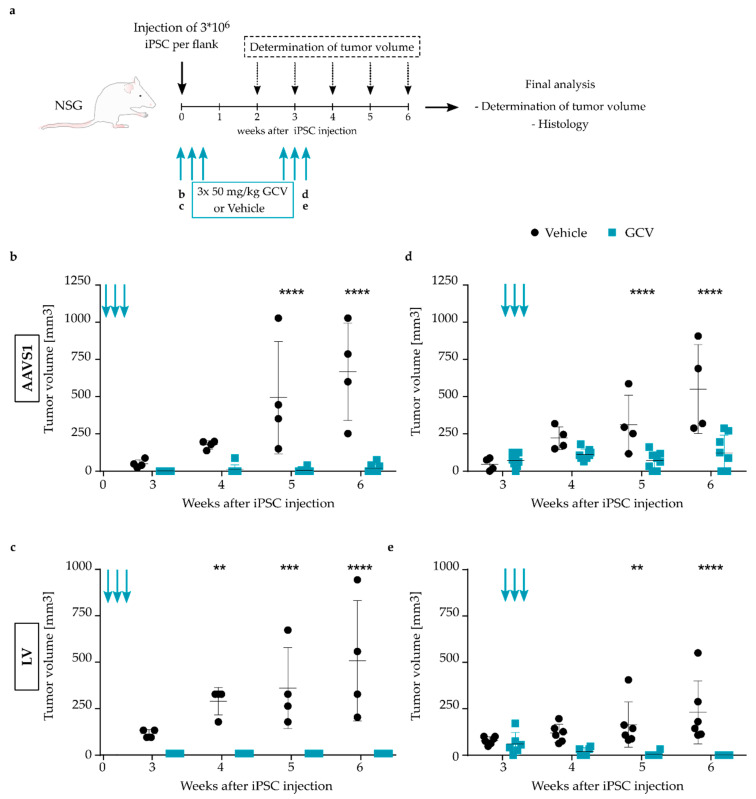
Efficient GCV-dependent in vivo ablation of TK.007 transgenic iPSC-derived tumors. (**a**) Schematic depiction of the experimental design to test TK.007/GCV-mediated in vivo ablation. NSG mice were injected with 3 * 10^6^ iPSCs per flank and treated either immediately or 2–3 weeks later with three rounds of injections (blue arrows) performed on consecutive days with 50 mg/kg GCV or vehicle (NaCl). The tumor diameter was determined weekly until end-point analysis at week 6, which additionally included analysis of tumor histology. (**b**,**c**) Tumor volume, weekly determined with a caliper, at the indicated time points after injection of AAVS1 CAGs.TK-transgenic (**b**) or LV CAGs.TK-transduced cells (**c**) into NSG mice. GCV or NaCl was administered immediately upon iPSC injection. Each data point represents one teratoma. N = 4–8. Arrows indicate the time points of GCV/NaCl treatment. (**d**,**e**) Tumor volume at the indicated time points after injection of AAVS1 CAGs.TK-transgenic (**d**) or LV CAGs.TK-transduced (**e**) cells into NSG mice. GCV or NaCl was administered upon emergence of palpable tumors. Each data point represents one teratoma. N = 4–6. ** *p* ≤ 0.01, *** *p* ≤ 0.001, **** *p* ≤ 0.0001.

As the safety switch concept is intended for use in clinical application of iPSC-derived cell grafts, we next validated the performance of the TK.007 safety switch in cells modified through AAVS1 targeting or LV vector transduction in a murine in vivo teratoma induction model. NSG mice were injected with AAVS1 CAGs.TK- or LV CAGs.TK-modified iPSCs in both flanks to induce teratoma formation (Figure 2a). In a first setting, the potential for TK.007-mediated prevention of teratoma formation was tested. Gene-modified cells were co-administered with 50 mg/kg GCV or vehicle (0.9% NaCl), followed by two further GCV/vehicle injections on the following two days. Tumor size was recorded weekly with a caliper, with tumors in vehicle-treated mice starting to emerge on both flanks from the third week onwards. In contrast, no teratoma formation was observed in GCV-treated mice at three weeks post-iPSC-application. Six weeks after the treatment, the mice were sacrificed and teratomas were dissected, revealing a significant difference in tumor size of vehicle-treated compared to GCV-treated mice, with only small tumor remnants present in the latter (Figure 2b,c). Histologic analysis confirmed the dissected teratomas of vehicle-treated mice to contain cells of all three germ layers (meso-, endo- and ectoderm, Figure S2a), whereas the tissue extracted from GCV-treated mice contained only adipose tissue remnants and thus did not represent teratoma formation (Figure S2b). In a second experimental setting, we aimed to analyze whether therapeutic treatment is also effective once teratomas are established. For this, it was assessed whether the growth of already established tumors derived from safety switch-equipped iPSCs could be halted or if tumor size could even be reduced upon GCV administration. The mice were treated with GCV or vehicle about three weeks post-iPSC-transplantation, once tumor nodules were palpable. As before, three repetitive administrations of GCV/vehicle were performed on consecutive days. Dissection of the teratomas was accomplished three weeks post-treatment. Demonstrating a therapeutic effect, GCV application to animals transplanted with *AAVS1*-modified cells resulted in a significantly lower tumor mass than seen in vehicle-treated animals (Figure 2d). Furthermore, the tumor volume in GCV-treated mice remained stable from the start of treatment onwards, with no further increase in tumor size until the end-point of analysis. Of note, the histologic characterization of some of the dissected tumors excised from GCV-treated mice revealed necrotic tissue and a strong infiltration of inflammatory cells (Figure S2c). Induction of the TK.007 safety switch system through GCV was also observed to halt or even reduce tumor size in animals transplanted with LV CAGs.TK-modified cells, confirming the suitability of the safety switch concept to inhibit tumor formation by transplanted pluripotent cells (Figure 2e).

Altogether, TK.007 expression from the CAGs promoter in combination with GCV treatment proved effective in eliminating transgenic cells in vitro and in vivo, and even blocked growth or resolved already established tumors. While equipment of employed cells with a functional safety switch expression cassette was possible both through TALEN-mediated integration into the *AAVS1* locus and through LV vector technology, stronger in vitro effects at low doses were observed with the latter, in line with the tendency for more pronounced in vivo effects.

### 3.3. Highly Efficient iC9 Safety Switch Eliminates Teratomas In Vivo

**Figure 3:**
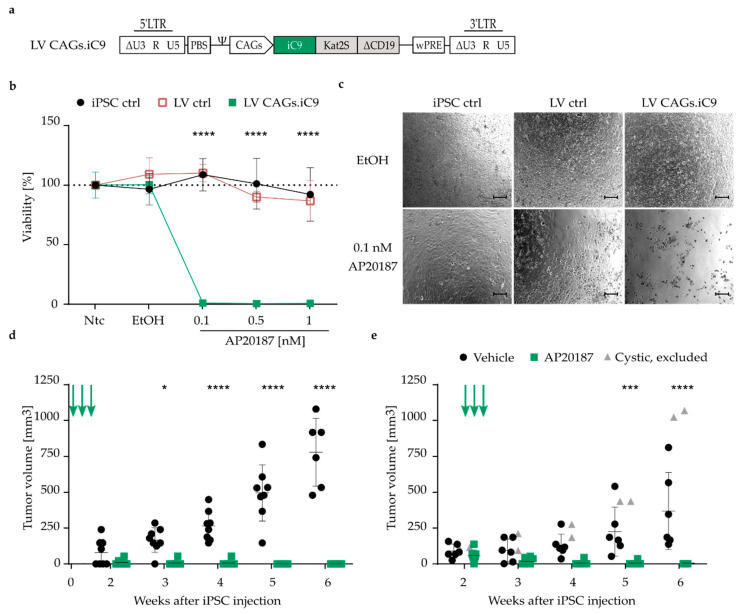
Efficient dimerizer-inducible in vitro and in vivo ablation of transgenic cells expressing iC9. (**a**) Lentiviral vector for expression of the iC9 safety switch, as well as the Kat2S and ΔCD19 markers under control of the CAGs promoter. LTR = long terminal repeat; ∆U3 = deleted enhancer/promoter region; R = repeat region within the LTR; Ψ = PSI, packaging signal; PBS = primer binding site; wPRE = woodchuck post-transcriptional regulatory element. (**b**) Percentage of viability relative to non-treated cells (Ntc) as determined by CellTiterGlo^®^ 2.0 cell viability assay. Unmodified iPSC (iPSC ctrl) and transgenic iPSC (LV ctrl, LV CAGs.iC9) were treated for 24 h with the dimerizer AP20187 at the indicated concentrations, treated with the vehicle (EtOH) only, or left untreated (non-treated control, Ntc) LV ctrl: N = 3, iPSC ctrl N = 7, LV CAGs.iC9 N = 9. (**c**) Microscopy images of the indicated iPSC cultures treated with vehicle (EtOH) or with 0.1 nM AP20187 upon 24h of treatment. Scale bar: 100 µm. (**d**) Tumor volume, determined weekly with a caliper, at the indicated time points after injection of LV CAGs.iC9-transduced iPSCs into NSG mice. AP20187 or vehicle (AP20187-buffer) was administered immediately upon iPSC injection. Each data point represents one teratoma. N = 8; one mouse of the vehicle-treated group died in week 6. Arrows indicate the time points of AP20187/vehicle (AP20187-buffer) treatment. (**e**) Tumor volume at the indicated time points after injection of LV CAGs.iC9-transduced iPSCs into NSG mice. AP20187 or AP20187-buffer was administered upon emergence of palpable tumors. Each data point represents one teratoma. N = 6-8. Arrows indicate the time points of AP20187/vehicle (AP20187-buffer) treatment. Cystic tumors (grey triangles) were excluded from the analysis. * *p* ≤ 0.05, *** *p* ≤ 0.001, **** *p* ≤ 0.0001.

In addition to different concepts for genetic manipulation to install an expression cassette, different genetic suicide switches are available to achieve cell removal. The TK-system is attractive as it is already used in clinical applications [45]. However, its use is limited to proliferating cells, its viral origin could potentially induce immunogenicity and thus hamper successful transplantation, and GCV treatment of herpes simplex virus or cytomegalovirus infections would undesirably eliminate transgenic cells [46]. Therefore, we investigated the inducible caspase 9 (iC9) as a second strategy for the ablation of iPSCs and thereof derived cells. The iC9-based suicide system is known to have fast ablation kinetics induced upon the application of the chemical dimerizer AP20187, and is less immunogenic due to its human origin [47]. As LV vector-mediated suicide switch integration was equally potent or even slightly superior to AAVS1 integration in the TK.007/GCV setting, LV transfer was chosen for comparison of the efficiency of iC9 versus TK.007 suicide switches. The same vector design was used as before (Figure 1b), with the TK.007 transgene exchanged for iC9 to allow expression of the iC9 suicide switch under control of the CAGs promoter (Figure 3a). After three rounds of single colony cloning, a culture with a mean VCN of 1.7 was chosen for further experiments (Figure S3a). Again, LV transduction did not impact iPSC pluripotency, as demonstrated by *OCT4*, *NANOG* and TRA-1-60 expression (Figure S3b,c). Flow cytometric analysis of Kat2S and ∆CD19 expression indicated that the transgene cassette was actively expressed (Figure S3c,d). After 24 h of incubation with the dimerizer AP20187, more than 99% of iC9 transgenic cells (LV CAGs.iC9) were already eliminated at the lowest concentration tested (0.1 nM) (Figure 3b,c), whereas unmodified iPSCs (iPSC ctrl) and cells transduced with the control vector (LV ctrl) were insensitive towards the compound even at the highest concentration used in this setting (Figure 3b). In previous experiments, we observed that 60% of iC9 transgenic cells undergo apoptosis as early as 2 h after treatment with 0.5 nM AP20187. In contrast, for TK.007-modified cells, an incubation period of 72 h was required to reduce cell viability by 50% (data not shown). These criteria, i.e., the fast ablation kinetics, the less immunogenic human origin, and its use in clinics and the potent ablation in our setting, encouraged us to work with the iC9 safety switch system in all further experiments.

For in vivo assessment of the functionality of iC9-mediated prevention of tumor formation upon co-administration of the inducer and the halt of tumor growth or reduction of tumor size when therapeutically administering the inducer to mice with established tumors, the same experimental setting was followed as before (Figure 2a). When NSG mice were injected with LV CAGs.iC9 iPSCs into both flanks and immediately treated with the dimerizer, tumor formation was completely prevented (Figure 3d), whereas full teratomas containing cells of all three germ layers (Figure S3e) emerged in the vehicle (AP20187-buffer)-treated mice at two to three weeks post iPSC injection. Demonstrating a strong effect, we could successfully eliminate all tumors that had developed in the therapeutic setting where three repetitive injections of the dimerizer were applied when tumors were already palpable. Within one week, the tumor volumes of all mice were significantly reduced to a size that was no longer palpable (Figure 3e). In line with this observation, resection of the tissue in week six post iPSC injection only showed adipose tissue remnants, whereas mice treated with vehicle carried fulminant tumors representing cells of all three germ layers (Figure S3e).

### 3.4. Pluripotency-Specific Promoters Restrict the Effect of the Suicide Switch to iPSCs

**Figure 4:**
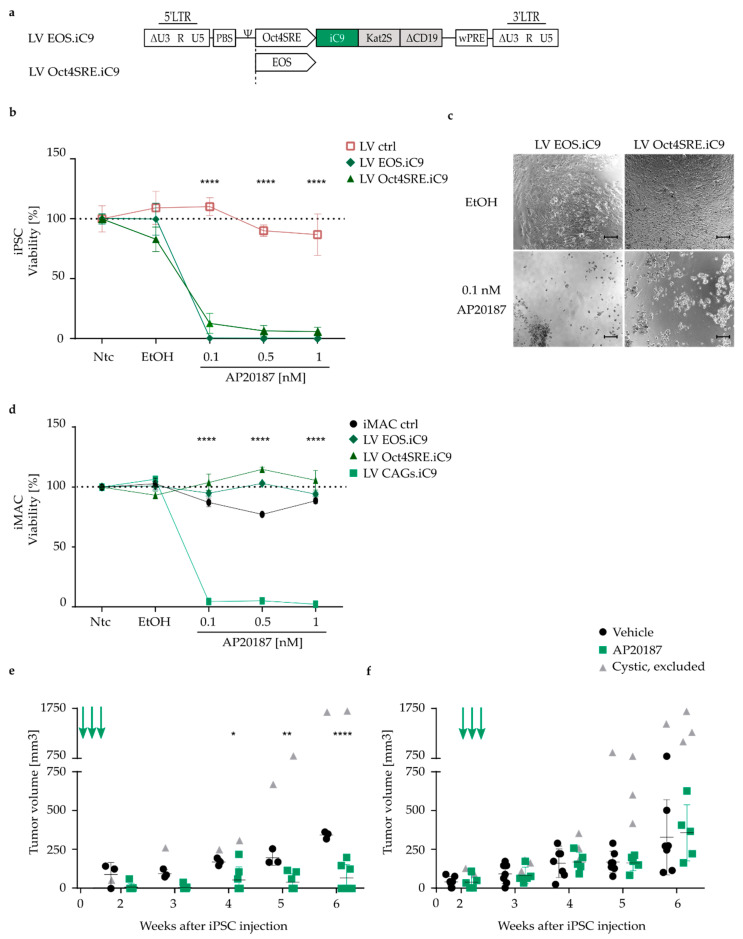
Promoter-dependent iC9-mediated selective ablation of iPSCs. (**a**) Lentiviral vectors using two different promoters, i.e., either the EOS (LV EOS.iC9) or the human Oct4SRE (LV Oct4SRE.iC9), for pluripotency-specific expression of the iC9, Kat2S and ΔCD19 transgenes. LTR = long terminal repeat; ∆U3 = deleted enhancer/promoter region; R = repeat region within the LTR; Ψ = PSI, packaging signal; PBS = primer binding site; wPRE = woodchuck post-transcriptional regulatory element. (**b**) Percentage of viability relative to non-treated control cells (Ntc) as determined by CellTiterGlo^®^ 2.0 cell viability assay. Unmodified iPSCs (iPSC ctrl) and transgenic iPSCs (LV ctrl, LV EOS.iC9, LV Oct4SRE.iC9) were treated for 24 h with the dimerizer AP20187 at the indicated concentrations, with vehicle (EtOH) only or left untreated (Ntc). N = 5. (**c**) Microscopy images of the indicated iPSC cultures treated with vehicle (EtOH) or with 0.1 nM AP20187 upon 24 h of treatment. Scale bar: 100 µm. (**d**) Percentage of viability of iMAC relative to non-treated cells as determined by CellTiterGlo^®^ 2.0 cell viability assay. Unmodified induced macrophages (iMAC ctrl) and transgenic iMAC (LV EOS.iC9, LV Oct4SRE.iC9, LV CAGs.iC9) were treated for 24 h with the dimerizer AP20187 at the indicated concentrations with the vehicle (EtOH) only or left untreated (Ntc). N = 3. (**e**) Tumor volume, determined with a caliper weekly, at the indicated time points after injection of LV Oct4SRE.iC9-transduced iPSCs into NSG mice. AP20187 or vehicle (AP20187-buffer) was administered immediately upon iPSC injection. Each data point represents one teratoma. N = 3–7. Arrows indicate the time points of AP20187/vehicle (AP20187-buffer) treatment. Cystic tumors (grey triangles) were excluded from the analysis. (**f**) Tumor volume at the indicated time points after injection of LV Oct4SRE.iC9-transduced iPSCs into NSG mice. AP20187 or AP20187-buffer was administered upon emergence of palpable tumors. Each data point represents one teratoma. N = 5–7. Arrows indicate the time points of AP20187/vehicle (AP20187-buffer) treatment. Cystic tumors (grey triangles) were excluded from the analysis. * *p* ≤ 0.05, ** *p* ≤ 0.01, **** *p* ≤ 0.0001.

Expression of the TK.007 and iC9 suicide switches under control of the CAGs promoter robustly and efficiently ablated transgenic cells both in vitro and in vivo upon administration of the corresponding prodrug or inducer, respectively. However, for future translation of iPSC-derived cell transplants and in a clinical context, elimination of the entire graft upon potential encounter of SAE may not be desirable, but rather to only eliminate the teratoma-forming cells. In addition, it could be envisioned to ablate all pluripotent cells remaining in the culture upon targeted differentiation into the desired cell type prior to transplantation of the graft. For this purpose, we tested the suitability of two different pluripotency-specific promoters, i.e., the Oct4SRE and EOS, to restrict suicide switch expression to pluripotent cells. LV vectors were used to integrate cassettes driving iC9 expression from the Oct4SRE or EOS promoter into iPSCs (Figure 4a), as this combination of transfer technology and suicide switch was identified as superior among the tested combinations in the previous experiments. In both cases, transduced cell clones chosen for all further experiments harbored a comparable VCN of 3 (Figure S4a) and transduction of iPSC cultures with the LV vectors (LV EOS.iC9, LV Oct4SRE.iC9) did not impact pluripotency as indicated by *OCT4*, *NANOG* and TRA-1-60 expression (Figure S4b,c). Indicating activity of the promoters in pluripotent cells, the fluorescent protein Kat2S and the surface marker ΔCD19 encoded on the vectors were robustly expressed in iPSCs, as confirmed by flow cytometric analysis (Figure S4c,d). Furthermore, efficient in vitro elimination of transgenic iPSCs was observed upon treatment with different concentrations of AP20187, with less than 5% of cells surviving a 24 h treatment at the lowest concentration (0.1 nM) of AP20187 tested (Figure 4b,c).

To confirm the restriction of Oct4SRE and EOS promoter activity to pluripotent cells, the LV vector-transduced iPSCs were differentiated towards the myeloid lineage using an embryoid body-based method [41]. The differentiated cells obtained a macrophage-like phenotype (iPSC-derived macrophages; iMAC) as indicated by flow cytometric analysis of their surface marker expression profile, with positive signals for CD45, CD11b, CD14 and CD163 markers (Figure S4e). In addition, cytospin analysis confirmed a macrophage-like cell morphology (Figure S4f). Promoter specificity was indicated, as iMAC carrying the LV Oct4SRE.iC9 or the LV EOS.iC9 vectors were insensitive to the treatment with different amounts of AP20187, with all cells surviving even at the highest drug concentration applied. In contrast, iMAC employing the CAGs promoter to drive iC9 expression were efficiently ablated upon AP20187 treatment (Figure 4d).

No significant differences were observed among the EOS and the Oct4SRE performance in terms of iC9 expression for in vitro elimination of transgenic cells. Therefore, to test the effects of iC9 expression under control of a pluripotency-specific promoter in the primary murine teratoma-ablation model, only iPSCs modified with the LV Oct4SRE.iC9 vector were injected, following the experimental setup as described before (Figure 2a). Mice that were treated with the dimerizer immediately upon cell injection demonstrated no or a significantly delayed tumor development compared to vehicle-treated mice (Figure 4e). In the therapeutic setting, mice that were treated with the dimerizer or vehicle once tumor nodules were palpable showed no difference in tumor growth until the final time point of analysis at six weeks post-iPSC-injection (Figure 4f). Of note, teratomas harboring a cystic, fluid-filled consistency were excluded from the analysis, representing false-positive enhanced tumor growth (Figure S2d).

In conclusion, we demonstrated that both the Oct4SRE as well as the EOS promoters restrict transgene expression to pluripotent cells, whereas the differentiated progeny were protected against the effects of induction of the iC9 system through AP20187. Using this concept, we were able to delay tumor growth from Oct4SRE.iC9-iPSCs when administering the dimerizer immediately upon injection, while established tumors that had already developed could not be controlled through dimerizer treatment.

## 4. Discussion

iPSCs are widely used for in vitro disease modeling of inherited diseases to gain deeper insights into disease function and potential treatment options [17,48,49]. Due to their potential to differentiate into all somatic cell types of the body, iPSCs also appear as a highly promising source for cell therapy. Allogenic iPSC-derived cell products have been reported to evade T- and NK-cell provoked immune rejections [50], so that characterized iPSC lines could be established and used to treat affected patients without the need to generate autologous iPSCs for each patient. However, HLA-matched or HLA-depleted cells, harboring even reduced immunogenicity [51] would be preferable for a universal off the shelf-iPS cell line, alleviating the need for lifelong use of immunosuppressants [52]. Due to the unlimited proliferation capability of iPSC, such lines can be indefinitely expanded and could thus be used for the treatment of a large number of patients. To derive the required cell type to be transplanted, available iPSC differentiation protocols and subsequent cell purification methods have been significantly improved over the past decade. However, the potential risk of SAE or presence of residual undifferentiated cells, so-called teratoma initiating cells (TIC) in the graft, which can give rise to benign teratomas or even form malignant tumors, still remains one of the most pressing challenges for the clinical translation of iPSC-derived cell products for personalized therapy [53,54,55]. Therefore, although iPSCs are easily generated and are extensively studied, so far only few clinical applications of iPSC-derived cell products have been reported [3,4]. To help realize improved safety of this alternative cell source, we genetically modified iPSCs to express genes that allow the specific elimination of transgene-positive cells upon drug treatment and we characterized and compared the efficacy of different suicide switches, promoters for their expression and modes for genetic introduction of the respective expression cassettes. While individual parameters tested here were previously investigated and the individual elements were used by others [7,29,31,56], this is (at least to our knowledge) the first systematic comparison to define optimal settings and combinations of safety switch, promoter and delivery tool for future application of the safety switch concept in personalized medicine.

In our study, iPSCs were routinely cultured on murine embryonic feeder cells, and thus a cocktail of the two cell types was transplanted in the in vivo experiments. For clinical application, a feeder-free iPSC line would certainly be desirable to avoid any unwanted side effects potentially induced by murine feeder cell contamination. However, this can be circumvented, as appropriate protocols and reagents for feeder-free iPSC culture are widely available.

The iC9 and the optimized TK.007 both efficiently ablated gene-modified cells in the pluripotent state in vitro and in vivo. However, in direct comparison, the TK.007-safety switch has some disadvantages, with one of them being its non-human origin, which might potentially evoke immunogenicity [57]. Although TK.007 demonstrated a superior killing compared to other TK-variants, is was shown to also exhibit a high bystander effect, i.e., to kill neighboring unmodified cells [11,29]. Nevertheless, the latter characteristic could also represent an advantage, as it may potentially increase elimination of expanding solid tumors containing mixed cell populations (genetically modified and unmodified). Another potential obstacle is the restriction of the effects of the TK.007/GCV system to proliferating cells due to its mode of action, so that cell grafts composed of fully differentiated, non-proliferating cells (i.e., resting macrophages) are not expected to be affected by GCV-treatment. In this context, future studies need to address whether non-proliferating cells are of any concern upon transplantation, and thus, if strategies for their removal are required in case of SAE. However, our approach would allow elimination of these cells by an alternative approach using CD19 antibody-based therapy [58] in case of any SAE, as a truncated version of the CD19 surface protein is co-expressed from the transgene cassette [59]. In contrast to TK.007, the iC9/AP20187 system is independent of the cell cycle and also ablation kinetics are reported to be very fast [7]. Upon treatment with AP20187, cells carrying the transgene under control of the CAGs promoter were efficiently eliminated with the lowest concentration of AP20187 tested (0.1 nM), while vehicle-treated and unmodified cells survived the treatment in vitro. Moreover, demonstrating in vivo efficacy of our approach in a teratoma-induction model, tumor development was prevented when mice were treated with AP20187 at the time point of iPSC injection. Furthermore, established tumors could be purged to an undetectable level upon AP20187 treatment. To further test the iC9-safety switch in a clinically relevant setting, in which iPSC-derived cells will be transplanted, iPSCs were differentiated into macrophages and treated with the dimerizer to induce cell death. Indeed, here we were also able to eliminate CAGs.iC9-transgenic iPSC-derived iMAC in vitro.

Based on the introduction of specific promoters, different cell fractions within the graft/cell pool were targeted for suicide gene expression. The ubiquitously expressing CAGs promoter proved suitable for elimination of both iPSCs and their differentiated progeny, while safety switch expression could be restricted to pluripotent cells by driving expression via the pluripotency-specific promoters Oct4SRE or EOS. Hotta et al. were the first to demonstrate robust expression from the EOS promoter in pluripotent cells, but not their differentiated progeny [27]. Others subsequently demonstrated the efficient ablation of pluripotent cells with the safety switches used in this study [22,60]. Vega-Crespo et al. established the ablation of transgenic cells expressing TK from the Oct4SRE [28]. To our knowledge, we are the first to present data using Oct4SRE for iC9-induced iPSC ablation. Pluripotent iPSCs were efficiently eliminated with our LV EOS.iC9 and LV Oct4SRE.iC9 vectors in vitro. Importantly, as desired, both pluripotency-specific promoters were unable to drive iC9-expression in terminally differentiated iMAC or induce their ablation upon AP20187 treatment. This specificity for pluripotent cells was confirmed in a murine in vivo teratoma-induction model, where the growth of established LV Oct4SRE.iC9-iPSC-derived teratomas was unaffected by AP20187 treatment. The occurrence of teratomas in mice simultaneously receiving iPSCs and AP20187 was either entirely prevented, or tumor establishment was at least delayed. This could indicate that the term “TIC” needs to be more broadly defined, i.e., not only including highly pluripotent cells, but also non-pluripotent, highly proliferative progenitors. *OCT4* expression is known to be highly specific to pluripotent and immature cells [61]. Upon differentiation, *OCT4* expression decreases [62] and so does the expression of the transgene when driven by an Oct4 promoter. Hence, non-*OCT4*-expressing but proliferating progenitor cells would escape safety switch treatment and could still give rise to teratomas. In this context, it was shown that only 500–1000 pluripotent cells are needed to form a teratoma [63], a cell number that may remain in the graft as a result of insufficient purification of the cell population to be transplanted [64]. Furthermore, additional parameters appear to impact the risk of tumor formation. For example, it was shown that a loss of *pten* was linked to the emergence of highly aggressive TIC from murine ES cells [65]. CD30 expression was described as unique to malignantly transformed human ES cells [66] and is also expressed in iPSCs, thus this may be potentially useful for purification of differentiated cells prior to transplantation [67]. However, even though the Oct4SRE-driven suicide switch concept applied in vivo did not always completely prevent tumor formation, it might serve for the selective removal of pluripotent cells from a graft prior to its transplantation, applying the suicide concept as a step during the purification process of cells differentiated from iPSCs. Proof-of-concept for such a strategy was shown by Lim et al., who used the Oct4 promoter to drive safety switch expression for purification of a cell graft prior to transplantation [60]. Alternatively, in order to establish a concept for the in vivo removal of cells bearing the potential for tumor formation, promoters with activity that is less tightly associated to the pluripotent stage, but rather more widely given in diverse progenitor populations, may prove useful.

A very elegant solution to tackle the problem of TIC potentially present in iPSC-derived cell grafts would be the use of a bidirectional system allowing to select between ablation of only the pluripotent cells or of the entire graft. For this, different safety switches could be co-installed, driving one under control of a pluripotency-specific promoter and another driven by a ubiquitously active promoter, to allow selective elimination of the cell population by choice of the drug administered. First insights into the suitability of such an approach were provided by Loh et al., who co-integrated safety switches into pluripotency genes and a house-keeping gene using the CRISPR/Cas9 system [68].

In our study, two well-established and widely used modes for stable integration of an expression cassette into target cells (iPSCs) were compared in the context of introducing a genetic safety switch. In direct comparison, both methods allowed for stable genomic integration of the TK.007 and reporter transgenes into iPSCs and enabled a robust expression from the transgene cassettes. However, LV vector-modified iPSCs exhibited higher expression levels of the reporter transgene as compared to AAVS1-modified cells. This was despite the lower copy number of the installed cassette delivered via LV (VCN of 0.7) which was less than half of that installed in the biallelic targeted (i.e., harboring two copies per cell) AAVS1-modified cells. In line with this observation, AAVS1-modified cells were less efficiently eliminated in vitro upon 120 h of treatment with 0.1 µM GCV. Although AAVS1-modified cells stably expressed the transgene over time, there may be a fraction of cells in the culture in which the cassette inside the AAVS1 locus might have been silenced, as was seen in other studies [18,19]. A methylation analysis of the AAVS1 locus and the integrated donor cassette would elucidate if potential silencing occurred. Furthermore, additional iPSC clones would need to be analyzed in the LV vector setting to draw a general conclusion, as it has to be excluded that the specific integration site in the culture selected for validation of the suicide concept in this study is responsible for the high-level of transgene expression. The VCN of 0.7, however, indicates the LV-iPSCs to be non-clonal, despite low-density seeding and three rounds of single colony picking. In this context, it was shown that iPSCs with different vector integration sites form aggregated colonies after low-density seeding [69], possibly explaining the variable VCN in our study. Differences between AAVS1- and LV vector-modified iPSCs were also observed in an in vivo suicide switch-mediated tumor ablation model. Here, the size of teratomas grown from AAVS1 CAGs.TK-modified cells were efficiently reduced or tumor growth was at least partially inhibited upon GCV administration. However, the same treatment was sufficient to entirely prevent the development of teratomas in mice receiving LV CAGs.TK-iPSCs and to even eliminate already established teratomas. In contrast to our setting, complete elimination of the entire graft was not achieved in other studies, i.e., using LV vector TK-modified cells, although a higher number of repetitive GCV injections was applied, resistant clones that were insensitive towards GCV treatment were observed [56].

Targeting the AAVS1 safe harbor locus, which was accomplished using a designer nuclease (transcription activator like effector nuclease, TALEN), offers the advantage of selective integration into a defined locus, that is well-characterized as a suitable site for long-term transgene expression without side effects resulting from the introduction of foreign genetic material into this site. However, due to the risk of off-target integration of the donor, gene-modified cells have to be characterized for on- and off-target integrations. Of note, this is a minor challenge with iPSCs as target cells, as these (in contrast to certain differentiated cells and cells with limited expansion capacity) are compatible with single-cell cloning and unlimited expansion, so that stable clones can be generated, analyzed, selected and expanded for further usage. In contrast to targeted integration, the lentiviral vector system tested as a second transfer approach in our study exhibits a (semi) random integration profile. This offers easier handling, with no need for off-target screening, and also allows installing more than two copies of the expression cassette, if desired, simply by adjusting the number of vector particles applied per cell. On the other hand, due to the (semi) random integration pattern, the effects of vector integration may vary depending on the respective integration site in a certain clone/cell, and adverse effects resulting from vector-related insertional mutagenesis cannot be excluded. Therefore, we used an optimized SIN lentiviral vector platform with a low risk of causing insertional mutagenesis [21]. Furthermore, the efficiency of integration is much higher with the LV vector as compared to the TALEN-based AAVS1 integration platform.

Taken together, we provide proof-of-principle and compare efficacy and efficiency of genetic safety switches in vitro and in vivo as a basis for translation of iPSC-derived cell products into clinically applied, personalized treatment options.

## Data Availability

The datasets used and analyzed during the current study are available from the corresponding author upon reasonable request.

## Supplementary Materials

The following are available online at https://www.mdpi.com/article/10.3390/jpm11060565/s1, Figure S1: Characterization of AAVS1 CAGs.TK and LV CAGs.TK IPSC, Figure S2: Teratoma sections from AAVS1 CAGs.TK-iPSC and LV CAGs.TK-iPSC injected mice, Figure S3: Characterization of LV CAGs.iC9 cells, Figure S4: Characterization of LV Oct4SRE.iC9 and LV EOS.iC9 cells.
