# Supplementary material for: Efficient Genetic Safety Switches for Future Application of iPSC-Derived Cell Transplants
Source: J Pers Med. 2021 Jun 17;11(6):565. doi: 10.3390/jpm11060565 (PMC8234706; doi:10.3390/jpm11060565)
Supplement: Supplementary file 1 [file jpm-11-00565-s001.zip › jpm-1232780-supplementary.pdf]

Journal of *Personalized Medicine*

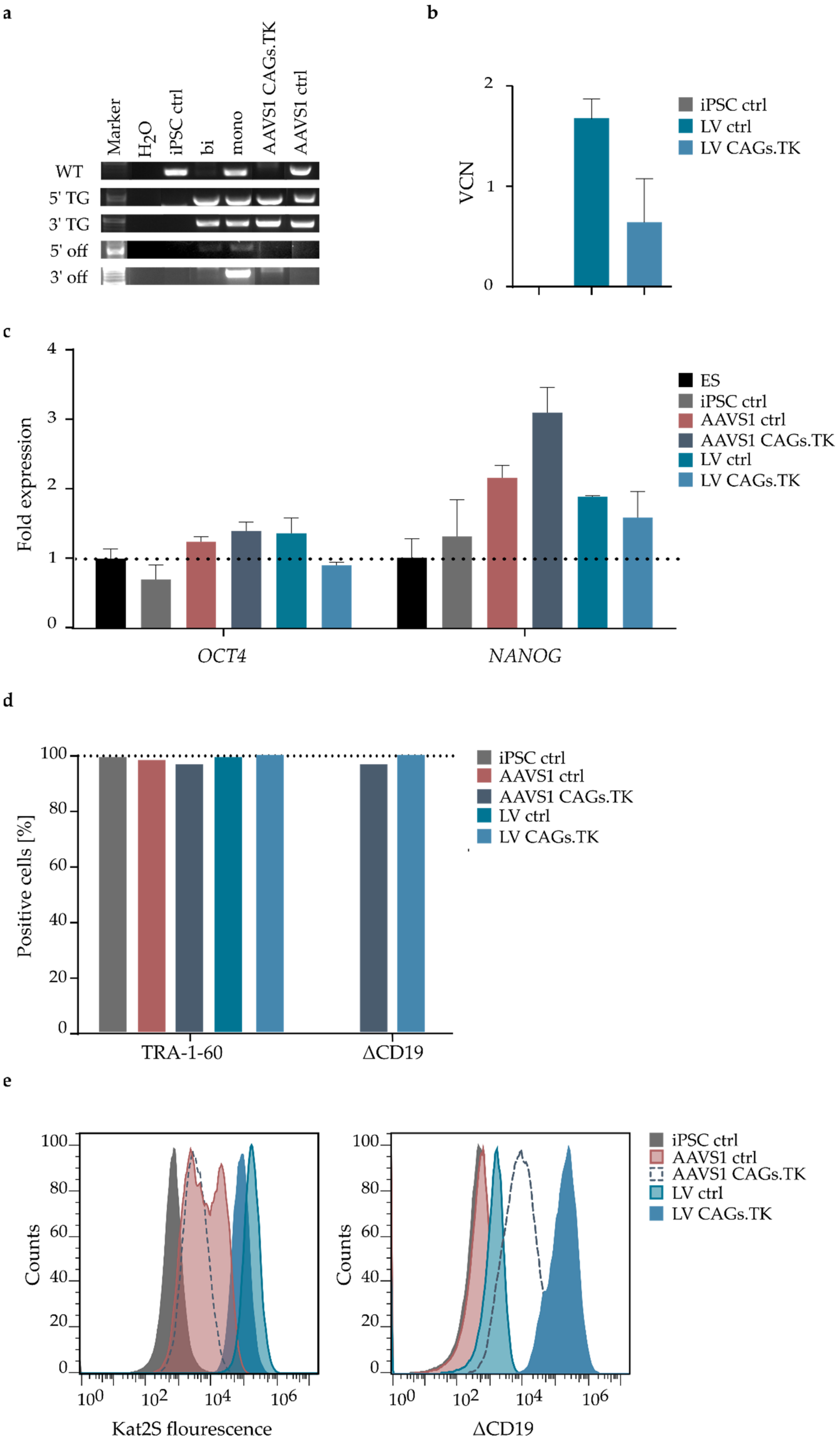

Figure S1. Characterization of AAVS1 CAGs.TK and LV CAGs.TK-IPSC. (**a**) PCR-based analysis of targeted integration into the AAVS1 locus. WT=Wild type; 5′TG= 5′ targeted integration; 3′TG= 3′ targeted integration; 5′off= 5′ off-target integration; 3′off= 3′ off-target integration; bi= biallelic-targeted control; mono= mono allelic-targeted control. (**b**) Vector copy number of transduced cells as determined by qPCR detecting wPRE. Technical replicates N=3. (**c**) RT-qPCR expression data of TK.007-modified cells for *NANOG* and *OCT4*. Expression levels are relative to H9 embryonic stem cells (ES). Technical replicates N=3. (**d**) Flow cytometry analysis of TRA-1-60 and ΔCD19 expression. Shown is the percentage of positive cells within the population of viable cells among 0.5-1*10^5 analyzed cells in total. (**e**) Flow cytometry analysis of Kat2S and ΔCD19 expression intensity in pluripotent TK.007-modified iPSC based on the analysis of a total of 0.5-1*10^5 cells.

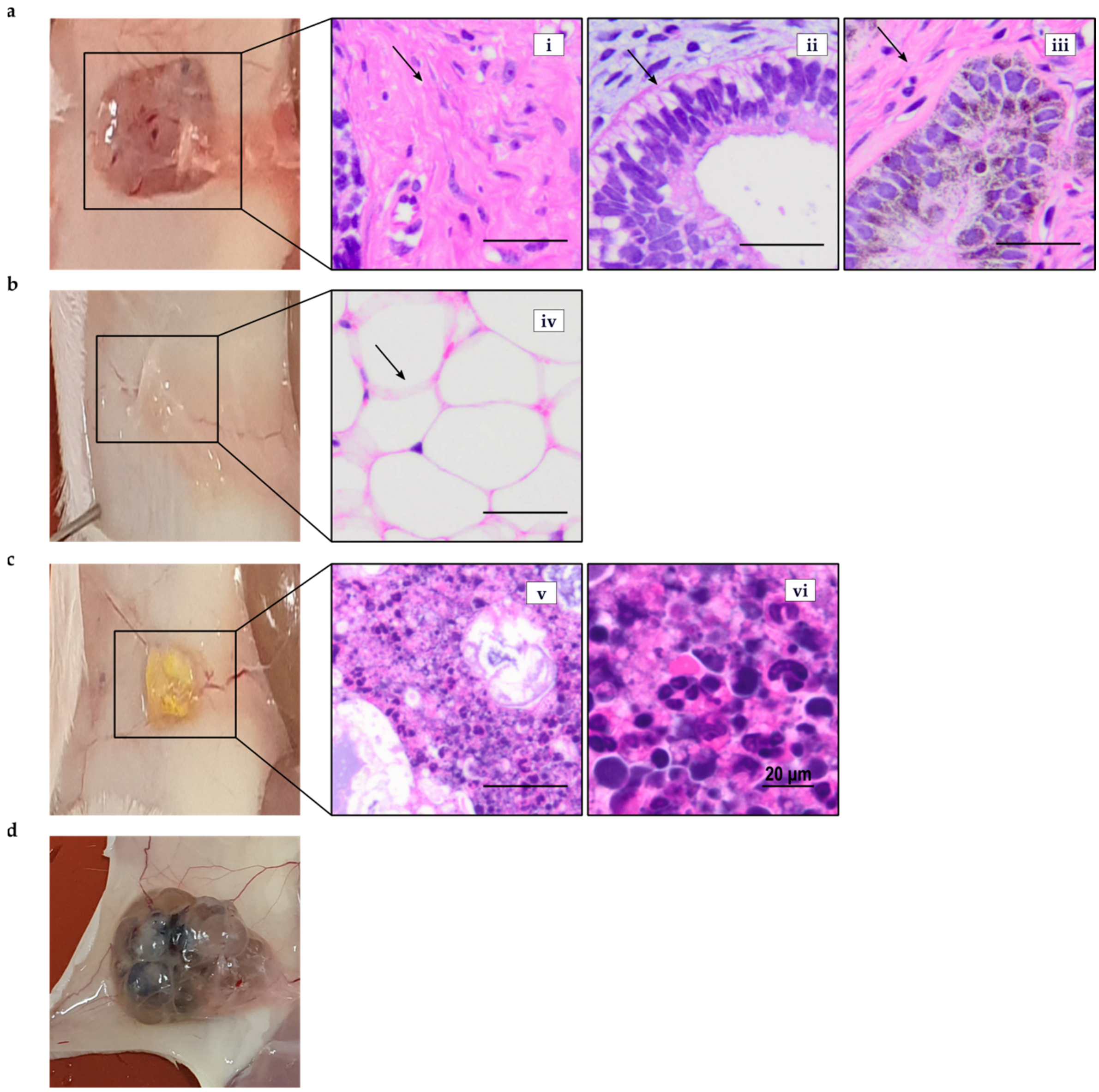

Figure S2. Teratoma sections from AAVS1 CAGs.TK-iPSC and LV CAGs.TK-iPSC injected mice. (**a**) Vehicle-treated teratomas, established from AAVS1 CAGs.TK and LV CAGs.TK iPSC are composed of all three germ layers (indicated by the arrows), mesoderm (muscular structure; i), endoderm (mucinous glandular epithelium; ii) and ectoderm (pigment epithelium; iii). (**b**) Animals that were immediately treated with GCV upon iPSC-injection demonstrated no palpable or visible teratomas. The tissue present at the injection site was adipose tissue (iv). (**c**) Animals treated with GCV upon tumor palpation demonstrated necrotic tissue remnants with infiltrating leukocytes at the former injection site (v, vi). (**d**) Exemplary picture of a cystic fluid-filled teratoma excluded from the analysis. Scale bar 100 µm (if not indicated otherwise).

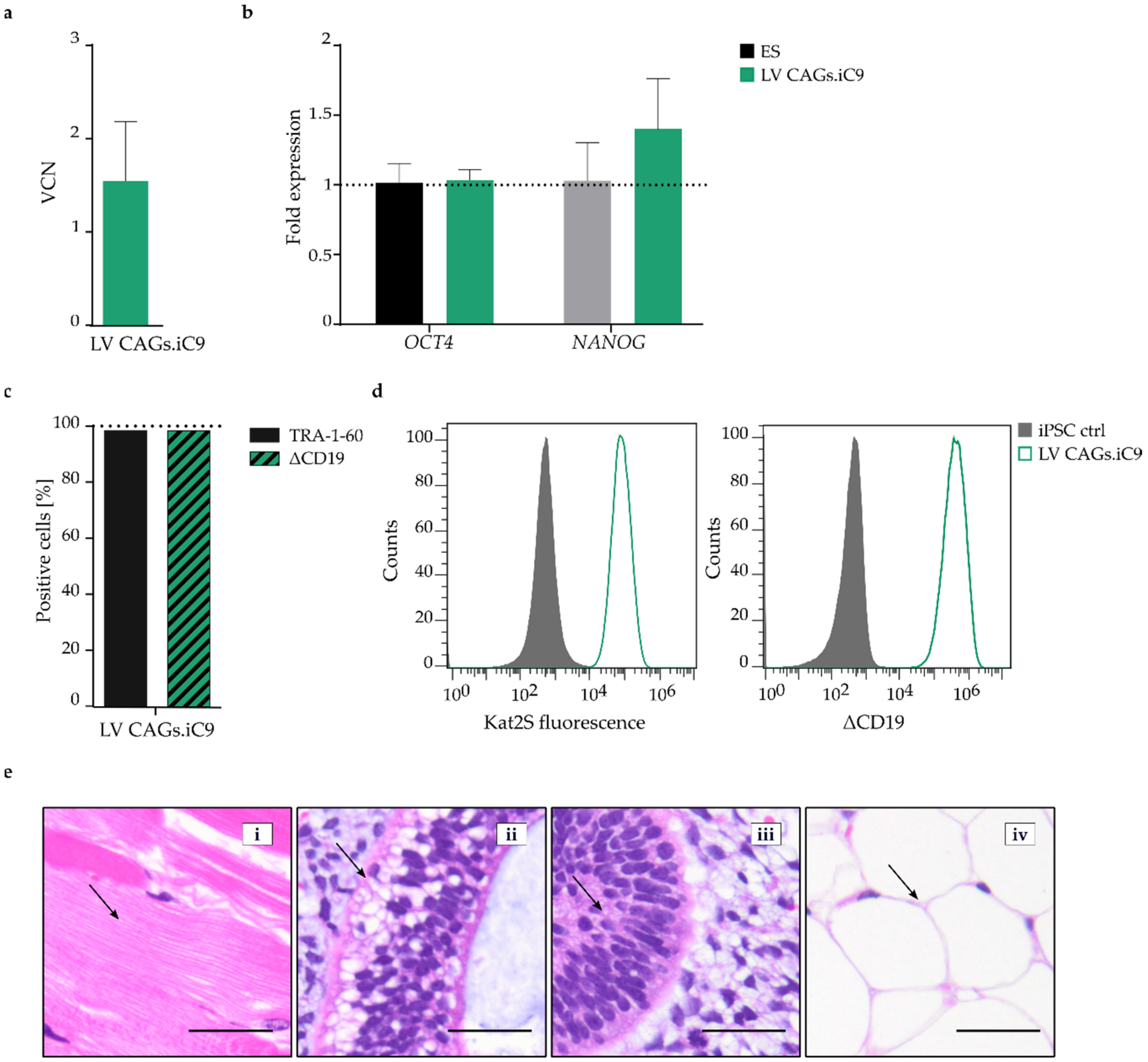

Figure S3. Characterization of LV CAGs.iC9 iPSC. (**a**) Vector copy number of transduced cells as determined by qPCR for wPRE. Technical replicates, N=3. (**b**) RT-qPCR analysis of *OCT4* and *NANOG* expression relative to embryonic stem cells (ES). Technical replicates N=3. (**c**) ΔCD19 and TRA-1-60 antibody staining analyzed by flow cytometry. Shown is the percentage of positive cells within the population of viable cells among 0.5-1*10^5 cells analyzed in total. (**d**) Kat2S and ΔCD19 expression intensity analyzed by flow cytometry using a total of 0.5-1*10^5 cells. (**e**) Sections of vehicle-treated LV CAGs.iC9 derived teratomas, representing cells of all three germ layers (indicated by the arrows), mesoderm (muscular structures; i), endoderm (mucinous glandular epithelium; ii) and ectoderm (neural rosette; iii). AP20187-treated animals demonstrated only adipose tissue remnants at the injection site (iv). Scale bar 100 µm.

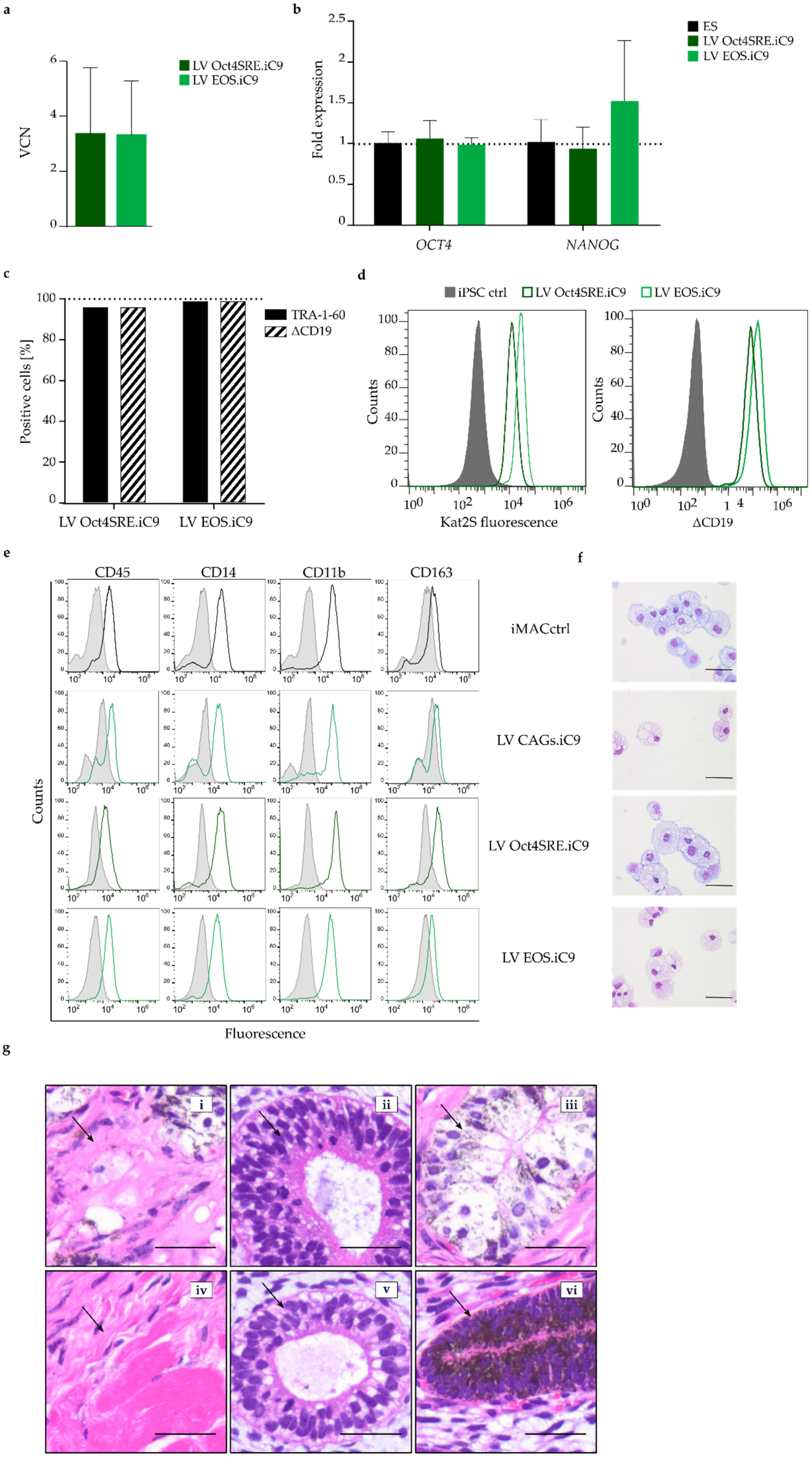
a
VCN
LV Oct4SRE.iC9
LV EOS.iC9
b
Fold expression
ES
LV Oct4SRE.iC9
LV EOS.iC9
OCT4
NANOG
c
Positive cells [%]
TRA-1-60
ΔCD19
LV Oct4SRE.iC9
LV EOS.iC9
d
iPSC ctrl
LV Oct4SRE.iC9
LV EOS.iC9
Counts
Kat2S fluorescence
ΔCD19
e
CD45
CD14
CD11b
CD163
Counts
Fluorescence
iMACctrl
LV CAGs.iC9
LV Oct4SRE.iC9
LV EOS.iC9
f
g
i
ii
iii
iv
v
vi

Figure S4. Characterization of LV Oct4SRE.iC9 and LV EOS.iC9 cells. (**a**) Vector copy number as determined by qPCR for wPRE. Technical replicates, N=3. (**b**) RT-qPCR analysis of *OCT4* and *NANOG* expression relative to embryonic stem cells (ES). Technical replicates N=3. (**c**) TRA-1-60 and ΔCD19 antibody staining analyzed by flow cytometry. Shown is the percentage of positive cells within the viable population among $0.5\text{-}1*10^5$ cells analyzed in total. (**d**) Kat2S and ΔCD19 expression intensity analyzed by flow cytometry using a total of $0.5\text{-}1*10^5$ cells. (**e**) Flow cytometry analysis of differentiated iMAC for expression of surface markers CD45, CD14, CD11b and CD163. Gray histograms indicate isotype control. Colored lines indicate stained cells. (**f**) Cytospin analysis of differentiated iMAC. Scale bar 50 μM. (**g**) Upper part: sections of vehicle-treated LV Oct4SRE.iC9 iPSC-derived teratomas, representing cells of all three germ layers (indicated by the arrows), i.e. mesoderm (muscular structures; i), endoderm (mucinous glandular epithelium; ii), ectoderm (pigment epithelium; iii); lower part: sections of AP20187-treated LV-Oct4SRE.iC9 iPSC-derived teratomas representing cells of all three germ layers (indicated by the arrows), i.e. mesoderm (muscular structures; iv), endoderm (mucinous glandular epithelium; v) and ectoderm (pigment epithelium; vi). Scale bar 100 μm.
